# proBDNF is modified by advanced glycation end products in Alzheimer’s disease and causes neuronal apoptosis by inducing p75 neurotrophin receptor processing

**DOI:** 10.1186/s13041-018-0411-6

**Published:** 2018-11-14

**Authors:** Catherine Fleitas, Gerard Piñol-Ripoll, Pau Marfull, Daniel Rocandio, Isidro Ferrer, Claire Rampon, Joaquim Egea, Carme Espinet

**Affiliations:** 1 Molecular Developmental Neurobiology Group, IRBLleida-UDL Rovira Roure 82, 25198 Lleida, Spain; 2 Departament de Patologia i Terapèutica Experimental, Universitat de Barcelona, Barcelona, Spain; 3 Centro de Investigación Biomédica en Red de Enfermedades Neurodegenerativas (CIBERNED), Hospitalet de Llobregat, Barcelona, Spain; 4 Unitat Trastorns Cognitius, IRBLleida-Hospital Universitari Santa Maria Lleida, Lleida, Spain; 5 Centre de Recherches sur la Cognition Animale (CRCA), Centre de Biologie Intégrative (CBI), Université de Toulouse, CNRS, UPS, 31062 Toulouse, France; 6 Serra Húnter fellow, Associate Professor, Generalitat de Catalunya, Barcelona, Spain

**Keywords:** Alzheimer’s disease, Biomarkers, BDNF, p75, proBDNF, Oxidative stress, Sortilin

## Abstract

Alzheimer disease (AD) is a complex pathology related to multiple causes including oxidative stress. Brain-derived neurotrophic factor (BDNF) is a neutrotrophic factor essential for the survival and differentiation of neurons and is considered a key target in the pathophysiology of various neurodegenerative diseases, as for example AD. Contrarily to BDNF, the precursor form of BDNF (proBDNF) induces apoptosis through the specific interaction with p75 and its co-receptor, Sortilin.

We used hippocampal tissue and cerebrospinal fluid from AD patients and controls. to study the localization and the levels of proBDNF, p75 and Sortilin as well as the post-traduccional modifications of proBDNF induced by Radical Oxygen Species, by immunofluorescence and Western blot. Differentiation and survival were assessed on differentiated mouse hippocampal neurons derived from postnatal neural stem cells from WT animals or from the transgenic AD animal model APP/PS1∆E9, based on mutations of familiar AD. In AD patients we observe a significative increase of proBDNF and Sortilin expression and a significative increase of the ratio proBDNF/BDNF in their cerebrospinal fluid compared to controls. In addition, the proBDNF of AD patients is modified by ROS-derived advanced glycation end products, which prevent the processing of the proBDNF to the mature BDNF, leading to an increase of pathogenicity and a decrease of trophic effects. The cerebrospinal fluid from AD patients, but not from controls, induces apoptosis in differentiated hippocampal neurons mainly by the action of AGE-modified proBDNF present in the cerebrospinal fluid of the patients. This effect is triggered by the activation and processing of p75 that stimulate the internalization of the intracellular domain (ICD) within the nucleus causing apoptosis. Induction of apoptosis and p75 ICD internalization by AD patients-derived proBDNF is further enhanced in neuron cultures from the AD model expressing the APP/PS1∆E9 transgene.

Our results indicate the importance of proBDNF neurotoxic signaling in AD pathology essentially by three mechanisms: i) by an increase of proBDNF stability due to ROS-induced post-traductional modifications; ii) by the increase of expression of the p75 co-receptor, Sortilin and iii) by the increase of the basal levels of p75 processing found in AD.

**Electronic supplementary material:**

The online version of this article (10.1186/s13041-018-0411-6) contains supplementary material, which is available to authorized users.

## Background

Alzheimer’s disease (AD) is a neurodegenerative disorder characterized by episodic memory decline at onset and deficits in multiple cortical functions in later stages. Neuropathology hallmarks are senile plaques and neurofibrillary tangles accompanied by deficits in axonal transport, synaptic dysfunction, and neuronal loss. The exact molecular mechanisms that trigger AD pathogenesis and disease course are not entirely understood. Among them, the increase of oxidative stress related to aging seems to play a central role, not only in AD but also in other neurodegenerative diseases [1]. There has been much research into the possible involvement of neurotrophins in AD pathogenesis, including Nerve Growth factor (NGF) and Brain Derived Neurotrophic Factor (BDNF) [2]. This interest was further supported by studies demonstrating the neuroprotective activity against neurodegeneration in different animal models of several neurodegenerative diseases, including AD, which suggested therefore a promising therapeutic role for neurotrophins [3].

BDNF binds to TrkB tyrosine kinase receptor to trigger the neurotrophic signaling including neuron survival, differentiation and synaptic plasticity of various nerve cell populations during normal development and during tissue repair after injury [4, 5]. BDNF is synthesized as a precursor form (proBDNF) that is processed by the activity of several proteases rendering three main products of 14 kDa (mature BDNF), 28 kDa and 34 kDa [6]. ProBDNF was reported to be biologically active and, unlike BDNF, it was shown to induce apoptosis through its interaction with p75 and its co-receptor, Sortilin [6–11]. p75 belongs to the family of tumor necrosis factor receptors, has not catalytic activity but a conserved non functional death domain [12]. The signaling mechanisms of p75 are complex and involve several intracellular interactors which trigger specific effects including Sc1, NRAGE and Necdine (cell cycle arrest) [13], RhoA (neuritogenesis) [12] and NRIF (apoptosis) [14]. An important signaling event for p75 involved in apoptosis is the processing of the receptor by convertases, in particular by a α-protease (BACE1) followed by a γ-secretase activity (presenilin 1 or PS1), that yield a 20 kDa intracellular domain (p75ICD) that is translocated into the nucleus [15–18]. p75ICD regulates the activity of the transcription factor NRIF inducing apoptosis [19]. Besides all these functions, p75 has also been reported to act as a co-receptor for Trk, indicating that p75 plays a central role in integrating the neurotrophic effects triggered by neurotrophins (as Trk co-receptor) and the pro-apoptotic signaling triggered by pro-neurotrophins (as Sortilin co-receptor).

In normal brains, the pro-apoptotic function of pro-neurotrophins and the neurotrophic effects of mature neurotrophins are tightly controlled by their expression levels as well as by the expression levels of their receptors, in particular p75 which is crucial for the integration of the two effects. It is conceivable therefore that neurodegeneration during AD might be caused by an imbalance of survival and death mechanisms due to changes in the relative levels of these proteins. Accordingly, the cerebral cortex of AD patients showed increased levels of p75 [20, 21] concomitant with decreased levels of Trk receptors which will favor an apoptotic signaling [22–24]. BDNF signaling was also shown to be altered in AD as an splicing form of TrKB, which gives rise to a truncated form of the receptor lacking the tyrosine kinase domain (presumably acting as a dominant negative), is expressed predominantly in hippocampus and frontal cortex in AD [23]. On the other hand, several works have described a reduced concentration of BDNF in the CSF of AD-affected patients [24]. Paradoxically, some authors have shown a decrease of proBDNF expression in AD-affected human brains [25, 26]. However, in the CSF of AD-affected patients, the levels of proBDNF have not been analyzed yet. Moreover, there is no data in the literature about the relative levels of proBDNF and BDNF in the CSF of AD-affected individuals compared to controls.

The relative levels of proBDNF and BDNF are directly regulated by the activity of the proteases involved in their processing. We have previously shown that proNGF extracted from brains of AD patients was more resistant to be processed by convertases in comparison to the proNGF isolated from control brains [27, 28]. We reported that the relative stability of proNGF in AD brains was due to post-traduccional modifications as a consequence of an increase of oxidative stress in the affected tissue [28]. In particular, we observed that Glyoxal (GO) and methylglyoxal (MGO), two highly reactive dicarbonyls that are increased during OS, reacted with free amino groups of Lys, Arg and Cys residues in proNGF, leading to the formation of the AGE/ALE adducts Nε-(carboxymethyl)-lysine (CML), Nε-(carboxyethyl)-lysine (CEL) and intermolecular crosslink and making proNGF in AD patients more resistant to the action of convertases [28].

In the present work we have studied the relative levels of BDNF and proBDNF and their receptors in AD patients and found a significant increase of Sortilin and proBDNF in the hippocampus. We also detected a significant increase of the ratio proBDNF/BDNF in the CSF of the AD patients, which shows a good correlation with the pathogenic effect of the pro-neurotrophin in the brain. Interestingly, the proBDNF in the CSF of AD patients showed an increase of CEL modifications which account for the increase of the proBDNF/BDNF ratio due to the major stability of the modified pro-form. Stimulation of primary cultures of hippocampal neurons with CSF from AD patients increased significantly the apoptotic cell death and the nuclear localization of p75ICD compared with the CSF from controls. Similar effects were observed when neurons were stimulated with a proBDNF that was modified in vitro by MGO. Importantly apoptosis and p75ICD nuclear localization were strongly reduced when the CSF of AD patients was immunodepleted of proBDNF, indicating that these effects were mediated, to a large extent, by the presence of proBDNF in the CSF. Finally, we demonstrated that the modified proBDNF had a stronger effect on apoptosis and p75ICD nuclear localization on primary cultures of hippocampal neurons obtained from a transgenic AD mouse model expressing the APP/PS1∆E9 transgene. In summary, we propose that in AD, proBDNF-p75/Sortilin signaling has an important contribution to the pathogenesis of the disease, causing an increase of cell death and impairing neuronal differentiation.

## Methods

### Human brain samples

Brain samples were obtained from the Institute of Neuropathology, Bellvitge University Hospital. Brain tissue was obtained from the Institute of Neuropathology HUB-ICO-IDIBELL Biobank and the Hospital Clinic-IDIBAPS Biobank following the guidelines of Spanish legislation on this matter (Real Decreto de Biobancos 1716/2011) and approval of the local ethics committees. At autopsy, one hemisphere was rapidly cut in coronal sections 1 cm thick and selected areas of the encephalon were dissected, frozen on dry ice, and stored at − 80 °C in labeled plastic bags until use. The other hemisphere was fixed by immersion in 4% buffered formalin for three weeks for morphologic examination. The neuropathological study was carried out on twenty-five regions of the cerebral cortex, diencephalon, thalamus, brainstem, and cerebellum. De-waxed paraffin sections were stained with hematoxylin and eosin and Klüver-Barrera and processed for immunohistochemistry to microglia-specific markers, glial fibrillary acidic protein, β-amyloid, phosphorylated tau, α-synuclein, TDP-43, ubiquitin, and p62. Neuropathological diagnosis of the AD was carried out following the Braak, and Braak stages [29] adapted to paraffin sections [30]. Cases with concomitant pathologies, including Lewy body diseases, tauopathies (particularly argyrophilic grain disease), vascular diseases, TDP-43pathies, and metabolic syndrome were excluded. Control and disease cases were processed in parallel. The anterior hippocampus area was used for further immunohistochemical studies.

### Human CSF samples

CSF samples were obtained from control and AD-affected patients and maintained at 4 °C for less than 4 h. All patients have signed informed consent and the study has been approved by the hospital’s ethics committee. All patients underwent lumbar puncture between 8:00 and 10:00 in the morning to avoid variations relating to the circadian rhythm. Protein concentration was determined by DC-Protein Assay (Bio-Rad). Samples were collected and frozen in polypropylene tubes. Post-puncture, CSF was centrifuged at 2000 g for 10 min at 4 °C and stored at − 80 °C until the use of the samples. The levels in CSF Aβ42, t-tau and p-tau were determined by a method of enzyme-immuno assay (ELISA) using the kit Innotest Amyliod β (1–42), Innotest htau and phospho-tau 181 (Innogenetics ®) according to the manufacturer’s instructions. All samples were measured in duplicate and were expressed as pg/ml. We use cut off points based on the calculation of sensitivity and specificity of our own study population (different from this sample).

### Animal model

APPswe/PS1DE9 with a C57Bl/6 background (double transgenic mice expressing a chimeric mouse/human amyloid precursor protein and a mutant human PS1 with deletion in exon 9) were purchased from The Jackson Laboratory and kept in specific pathogen free conditions under standard animal housing conditions in a 12-h dark–light cycle with free access to food and water in the animal house facility of the Universitat de Lleida [31, 32]. Heterozygous males were bred with wild-type C57/Bl6 females. Animal procedures were conducted according to ethical guidelines (European Communities Council Directive 86/609/EEC) and approved by the local ethics committee of the Universitat de Lleida. For experiments, tail biopsies were taken from P0 offsprings for genotyping by PCR according to the PCR conditions suggested by The Jackson Laboratory. Mice not expressing the transgene were used as controls.

### Cell culture

Hippocampal Neural Stem Cells (NSCs) were isolated from neonatal pups (P0–P1) from controls and APP/PS1 transgenic mice. Briefly, the brains were dissected in Hanks Medium 1X (Gibco 11,039–021) supplemented with Penicillin-Streptomycin (P/S) (1%) (Sigma-Aldrich P4333). After dissection, the hippocampi were washed with Hanks Medium with P/S (1%) and dissociated in Hanks Medium with Papain (Sigma-Aldrich 1,001,992,754) for 20 min at 37 °C. Papain activity was stopped with two washes with Trypsin inhibitor (10 mg/ml in Hanks medium) (Roche Diagnostics 10,109,886,001) plus three washes with 1 ml of Dulbecco’s modified Eagle’s medium (DMEM)/F12, supplemented with B27 (2%) (Gibco 12,587–010) and P/S. Mechanical dissociation was performed with a heat-rounded Pasteur pipette tip. Dissociated cells were centrifuged at 600 rpm for 4 min, and the pellet was resuspended in DMEM/F12 (37 °C) supplemented with B27 (2%), P/S(1%), Epidermal Growth Factor (EGF) (20 μg/ml) (Sigma-Aldrich E9644) and Fibroblast Growth Factor (FGF) (20 μg/ml) (Alomone Labs F-170). Cells were platted on bacterial, non-treated, P35 plates and incubated for four days at 37 °C. After four days, the supernatant was centrifuged at 600 rpm for 4 min, the supernatant was removed and the pellet was washed in complete medium DMEM/F12 (37 °C) supplemented with B27 (2%) and P/S (1%), without growth factors. Then the neurospheres were dissociated with a pipette in 500 μl of complete medium and determined the cell concentration by Trypan Blue dye exclusion. Cells were plated at 6000 cells/well concentration in 8-well on a glass slide coated with poly-D-lysine (Sigma-Aldrich P-7886) and laminin (Invitrogen 23,017–015) with complete medium (DMEM/F12, B27 (2%) and P/S (1%)). Neurons were treated at 1 DIV. Treatments were done after two washes with DMEM/F12 without B27. The treatments with neurotrophins and controls are done with DMEM/F12 containing 0,25% B27. For treatments with CSF, all the media is substituted by CSF from controls and AD with P/S (1%). After six days (7 DIV in total), were fixed for 30 min in 4% (*w*/*v*) paraformaldehyde (PFA)/PBS for immunofluorescence assays.

### Western blotting

CSF samples were obtained as described in 2.2., from control and AD-affected patients and maintained at 4 °C for less than 4 h. Previously to the western blotting, CSF samples were concentrated 20X using Amicon Ultra 10,000 MWCO (Ultracel Low Binding Cellulose) (Millipore). Protein concentration was determined by DC-Protein Assay (Bio-Rad). Proteins were separated by electrophoresis in 12% SDS-PAGE and transferred to Immobilon-P membranes (Millipore). Membranes were blocked for 1 h at room temperature in TBS-T (50 mM Tris, pH 8.0; 133 mM NaCl, 0.2% Tween 20) with 5% skimmed milk and incubated with primary antibodies against the mature BDNF (1:1000 in TBS-T (Alomone) at 4 °C overnight and with HRP-conjugated secondary antibodies (Jackson) (1:5000 in TBS-T) at room temperature for 1 h. Detection was performed using an ECL chemiluminescence system (Amersham-Pharmacia) following the manufacturer’s instructions. For densitometry analysis of the immunoreactive bands, we used ImageJ (http://rsbweb.nih.gov/ij/) with local background subtracted. For each sample, the relative abundance of each BDNF isoform was expressed as a ratio of that particular BDNF isoform related to the total BDNF signal (sum of proBDNF and mature BDNF). The data were shown as the mean ± standard deviation (SD). Differences between groups were calculated using a 2-tailed Student’s t-test.

### AGE modifications of recombinant proBDNF

Recombinant human proBDNF (Alomone) was modified by the reactive carbonyl specie MGO that react with free amino groups of Lys residues on proteins, leading to the formation of CEL adducts and intermolecular crosslinks [28]. The reactions were performed for 24 h at 37 °C by mixing 500 μg recombinant protein with 250 μl 50 μM GO or MGO and 250 μl 100 mM sodium phosphate. The modified proBDNF was dialyzed using Amicon Ultra-4 Centrifugal Filter Units (Millipore) to remove the excess of MGO and replace the solution with PBS.

### Immunohistochemistry

Microtome sections of fixed and paraffin-embedded human hippocampus 30 μm thick were processed free-floating, and collected in slides. Before immunodetection, they were deparaffined, and rehydrated using standard procedures. Epitope retrieval was performed by incubating the slides in 10 mM sodium citrate buffer (pH 6.0) for 20 min at 95-98 °C in a water bath. After washing with PBS at room temperature, sections were permeabilized with PBS with 0, 1% Triton X-100 (PBST) and blocked for 1 h at room temperature with 5% donkey serum in PSBT. Sections were incubated with primary antibodies in blocking solution over night at 4 °C (rabbit anti-proBDNF, 1:100 (Alomone); mouse anti-CEL, 1:100 (Trans Genic Inc); rabbit anti-ECDp75, 1:100 (Abcam); goat anti-Sortilin, 1:100 (R&D System)). After washing the sections were incubated for 2 h at room temperature with the corresponding secondary antibodies in blocking solution containing DAPI 1:2000 (donkey anti-goat Cy3, 1:500; donkey anti-rabbit Alexa488, 1:500; donkey anti-mouse Cy3, 1:500 (all from Jackson Immunoresearch). Blockage of anti-proBDNF antibody immunoreactivity was performed by incubating the antigenic peptide with the antibody in a proportion 10:1, during 2 h at room temperature, before the immunohistochemistry procedure. Fluorescence images were imaged on an Olympus Bx51 fluorescence microscope or under a Fluo View FV-1000 Olympus laser-scanning confocal microscope, and micrographs were uniformly adjusted for levels, brightness, and contrast in Adobe Photoshop.

### Immunocytochemistry

Cells were fixed with 4% PFA in PBS for 30 min at room temperature, permeabilized with TBST for 60 min at room temperature and blocked with 5% donkey serum in TBST. Primary antibodies were diluted in blocking solution and incubated overnight at 4 °C (rabbit anti-human p75, 1:100 (Promega) to detect intracellular domain, rabbit anti-ECD p75, 1:100 (Abcam) to detect the extracellular domain of p75NTR, goat anti-Sortilin, 1:100 (R&D System); rabbit anti-TrkB, 1:100 (H-181, Santa Cruz Biotech.); goat anti-SV2,1:100 (DSHB); mouse anti-βIII tubulin, 1:100 (Sigma); mouse anti-doublecortin, 1:100 (Cell Signaling), rabbit anti-calbindin, rabbit anti-calretinin and parvalbumin,1:100 (Swant)). After washing samples were incubated for 2 h at room temperature with the corresponding secondary antibodies diluted in blocking solution containing DAPI 1:2000 (monkey anti-goat Cy3, 1:500; donkey anti-rabbit Alexa488, 1:500; donkey anti-mouse Cy3, 1:500 (all from Jackson Immunoresearch)). Fluorescence images were acquired on a confocal microscopy setup (Olympus FV1000, 60X PlanApo using software Fluoview v.4.3) or on an inverted fluorescence microscope (OlimpusIX71, 20X LCPlanFl).

### Quantitative imaging

All microscope settings were set to collect images below saturation and were kept constant for all images taken in one experiment. Statistical significance (*p* values) was assessed using the two-tailed Student’s t-test unless otherwise indicated. Results were shown as the mean +/− standard deviation (SD).

### Statistical analysis

Statistical analysis of data was performed using SPSS statistical software (SPSS for Windows, v.16, SPSS, Inc., Chicago, IL). Student’s t-test and One-way ANOVA analysis are used to identify significant differences.

## Results

### proBDNF expression and the ratio Sortilin/p75 are increased in the hippocampus of AD patients

**Fig. 1:**
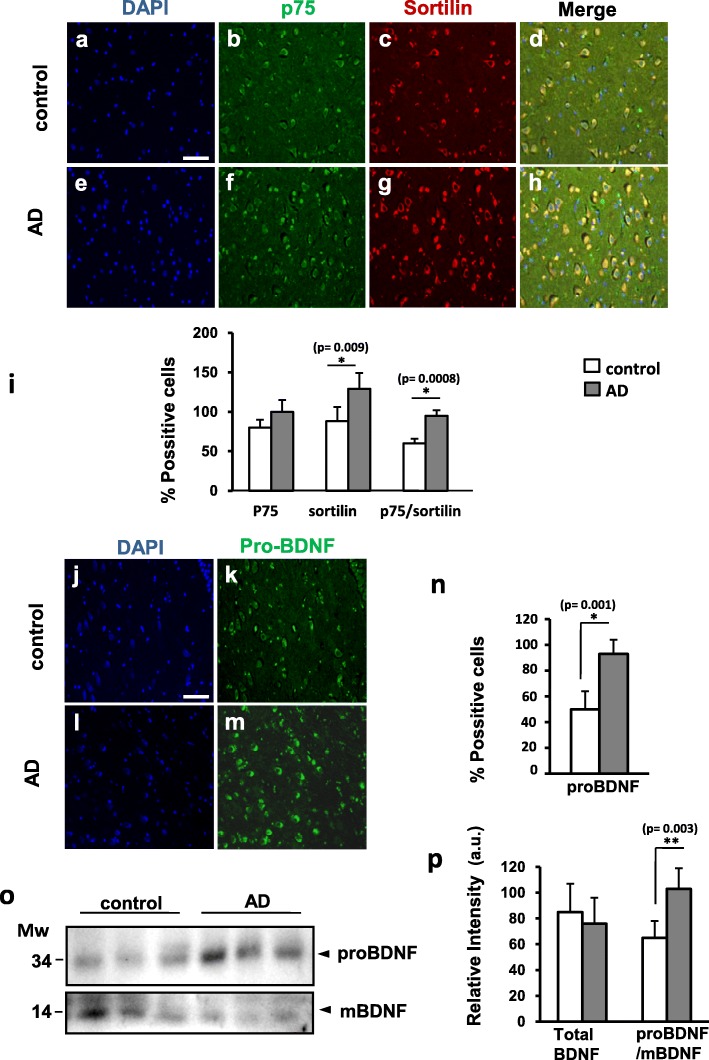
Sortilin and proBDNF expression is increased in the hippocampal hilus in human control and AD-affected cases. Representative immunofluorescence pictures stained with DAPI (**a, e, j, l**), ECDp75 (**b, f**), Sortilin (**c, g**) and proBDNF (**k, m**) at control and AD cases. **i)** Quantification of Sortilin immunoreactivity and p75/Sortilin co-localization in the AD is significantly increased respect to the controls. **n)** Quantification of proBDNF expression is significantly increased in the AD respect to the controls. Bars represent the mean of % positive cells ± SD. respect to DAPI stained cells. Positive cells are considered those with staining in the whole cell body. 300 total cells from each sample from 5 control and 5 AD-affected cases are counted. **o)** Western blotting analysis of BDNF forms in human CSF samples. The anti mBDNF antibody detect proBDNF (34 kDa) and mBDNF (14 kDa). Both panels correspond to the same WB. Lanes correspond to different representative individuals. **p)** Densitometry analysis of immunodetected bands shows a significant increase proBDNF/mBDNF ratio in AD-affected patients. Bars represent the mean of 9 control and 9 AD samples as the percentage of the highest value in each WB, corrected for the corresponding densitometric values of the Coomassie-stained membrane. Scale bar =50 μm. * *p* < 0,05, ** *p* < 0,01, two tailed Student’s “t” test

The hippocampus is one of the brain regions most affected in the AD [29, 30]. Particularly vulnerable are a group of neurons located in the hilus, also known as hilear Mossy cells [33, 34]. We obtained brain samples containing this region from AD patients and controls as summarized in Table 1 and using antibodies directed against the extracellular domain of p75 (p75ECD), Sortilin and the pro-domain of BDNF, we performed immunofluorescence assays to determine the levels of these proteins in the two groups. We observed that fluorescence intensity for p75 in individual hileal cells of AD brains was not significantly different on average compared to control brains (Fig. 1b, f, i). However, Sortilin fluorescence as well as the percentage of double positive cells for Sortilin and p75 increased significantly in AD brains (Fig. 1c, g, i). We also observed an abundant expression of proBDNF in the cytoplasm of the hileal neurons of human hippocampus (Fig. 1k, m).

**Table 1:** Summary of the patients from whom hippocampal samples were obtained and studied

N	Age	Gender	Diagnostic	Braak Stages	Post-mortem delay /hours
1	46	F	C	0	9
2	47	M	C	0	5
3	24	F	C	0	6
4	79	M	AD	V/B	5
5	82	F	AD	V/B	2
6	79	M	AD	V/C	7
7	85	F	AD	VI/C	12

*C* control, *AD* Alzheimer’s Disease, *A, B and C* Braak and Braak’s classification of AD stages depending on amyloid plaques, *0-V* Braak and Braak’s classification of AD stages depending on the distribution and amount of neurofibrillary tangles

However, in AD brain samples, fluorescence intensity of proBDNF was significantly higher than in control brains (Fig. 1k, m, n). Next we asked whether proBDNF and BDNF could be detected in the CSF of AD patients and controls since this technique has been traditionally used to study the expression profile of different markers in alive individuals for the diagnostic and/or the prognostic of neurodegenerative diseases. In this case, we analyzed the CSF by western blot and used antibodies against the mature form of BDNF that also detects the proBDNF. Levels of mature BDNF (14 kDa band) in CSF were very low compared to proBDNF (34 kDa band) and required longer exposure times that were shown in a separate panel (Fig. 1o). Densitometric analysis of the two bands in 15 AD-affected patients and 15 control individuals (see Table 2 summarizing the demographic and clinical characteristics of CSF donors) revealed that the total levels of BDNF (proBDNF+BDNF) were similar between the two groups (Fig. 1p). However, in AD patients we observed an increase of proBDNF associated with a decrease of BDNF, compared to controls (Fig. 1o). When we calculated the ratio proBDNF/BDNF in each sample, we observed that the average ratio proBDNF/BDNF was significantly higher in the CSF from AD patients (Fig. 1p). These results indicate that the CSF is a reliable sample that recapitulates the changes in proBDNF/BDNF expression that take place in the brain of AD patients. In summary, we conclude that hileal neurons of AD patients are more susceptible to cell death due to the higher levels of proBDNF and Sortilin expression and the higher proportion of cells expressing both p75 and Sortilin.

**Table 2:** Demographic and clinical characteristics of the patients from whom CSF samples were studied. Case (AD); control (C); *p* (Student’s t-test)

	Case(*n* = 15)	C(*n* = 15)	*P*
Male	4 (26.7%)	5 (33.3%)	0.7
Years	73.5 ± 12,1	70.5 ± 7,1	0.6
Age schooling	11.3 ± 5,0	11.3 ± 2,2	0.2
MMSE	19,1 ± 6,0	28,1 ± 1,8	0
**Family history**			
Presenilin AD	2(13.3%)	0(0%)	0.14
EA > 65 years	3(20.0%)	5(33.3%)	0.4
**Pathological history**			
Hypertension	6(40%)	8(53%)	0.46
Diabetes	3(20.0%)	2(13.3%)	0.6
Hypercolesterolemia	2(13.3%)	7(46.7%)	0.04
Depression	2(13.3%)	7(46.7%)	0.04
**CSF AD Biomarkers**	**pg/ml**	**pg/ml**	
Amyloid β	386,128	856 ± 204	0
Total Tau	615 ± 270	281 ± 103	0.01
PhosphoTau	85 ± 28	55 ± 18	0

### AGE modifications in proBDNF are increased in the hippocampus and the CSF of AD patients

**Fig. 2:**
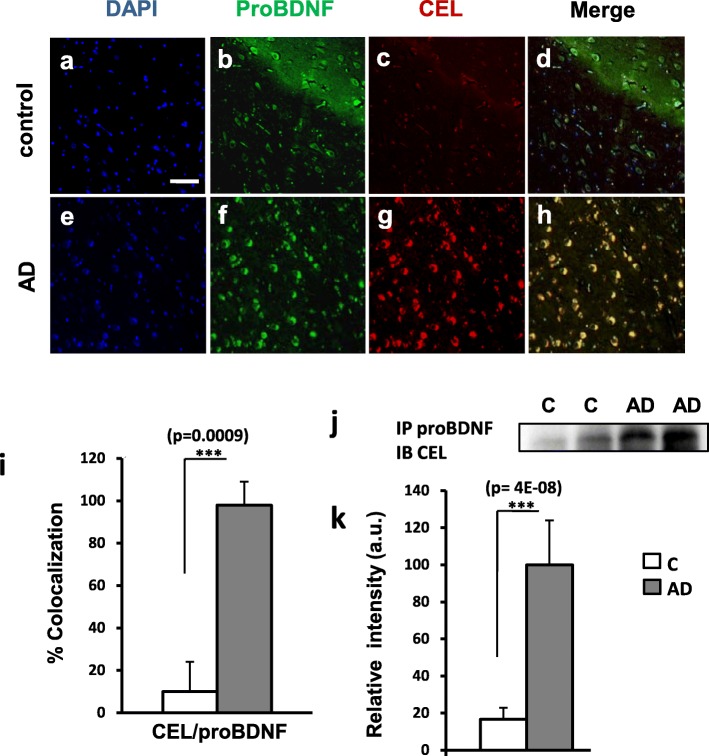
Hippocampal proBDNF is modiffied by AGE in AD human samples. Representative immunofluorescence pictures stained with DAPI (**a, e**), anti-proBDNF (**b, f**), anti-CEL (**c, g**) at control and AD cases. **i)** Quantification of CEL and proBDNF co-localization in the AD is significantly increased respect to the controls. Positive cells are considered those with staining in the whole cell body. Bars represent the mean of 5 control and 5 AD samples as % colocalization of CEL and proBDNF respect to the total cell number (DAPI staining) ± SD . 300 total cells from each sample are counted. **j)** Representative image of Immunoprecipitation of proBDNF from CSF from controls and AD patients analyzed by Western-blot with antibodies against CEL. **k)** Bars represent the mean of 9 control and 9 AD samples as the percentage of the highest value in each WB, corrected for the corresponding densitometric values of the Coomassie-stained membrane.Scale bar =50 μm . *** *p* < 0,001, two tailed Student’s “t” test

The increase of oxidative stress in the brain with aging has been proposed to be a key factor triggering and/or enhancing AD [1, 34]. The molecular mechanisms involved are diverse and include post-traductional modifications of specific proteins. For instance, GO and MGO, two highly reactive dicarbonyls that are increased during OS, react with free amino groups of Lys, Arg and Cys residues leading to the formation of the AGE/ALE adducts CML, CEL and intermolecular crosslink [35, 36]. As we have previously reported, these modifications affect proNGF during AD making the pro-neurotrophin form of NGF more stable (as it cannot be converted to mature NGF), which in turn increases cell death [28]. To determine whether proBDNF could be also modified by reactive dicarbonyls in AD, we performed different approaches. First, we studied the co-localization of proBDNF and CEL by immunohistochemistry in the hileal region of human hippocampal samples from controls and AD-affected brains. We observed a remarkable increase of CEL immunoreactivity in AD brains compared to controls (Fig. 2c, g). Interestingly, almost all the cells in the hillial region of AD patients with significant CEL staining, also co-expressed proBDNF, while in control brains very few neurons displayed double immunoreactivity (Fig. 2b-d, f-h, i). These results strongly suggested that proBDNF could be modified by reactive dicarbonyls. In order to address this question directly we performed a more specific assay. We immunoprecipitated proBDNF from the CSF of controls and AD patients and analyzed the precipitates by Western blot with an antibody against CEL. We found that the proBDNF in the CSF from AD patients showed a prominent CEL modification, around 6-fold on average, compared to controls (Fig. 2j, k). Altogether these results indicate that the proBDNF in AD patients displays AGE post-traduccional modifications as consequence of an increase of oxidative stress during neurodegeneration that might prevent the action of convertases to produce mature BDNF (see below).

### proBDNF modified in vitro by MGO induces neuronal apoptosis and decreases neuron differentiation

**Fig. 3:**
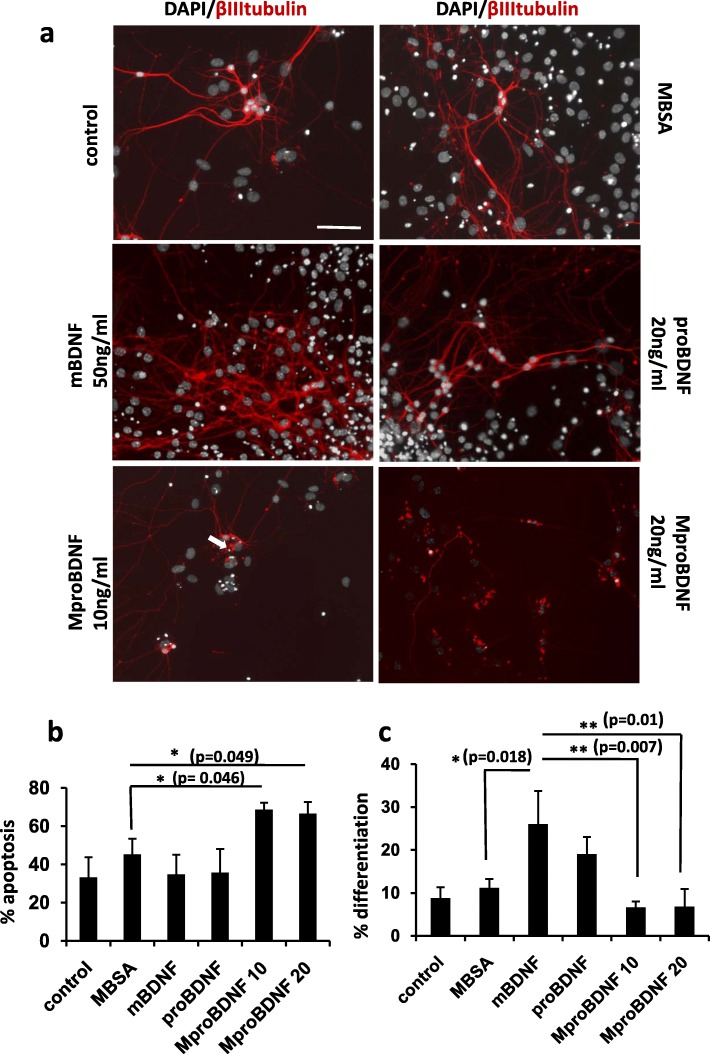
proBDNF modified by MGO induces apoptosis in control mice differentiated hippocampal neural stem cells . **a)** Representative pictures of neuronal primary culture treated for 6 days (7DIV) with: untreated (control), MGO modified BSA (MBSA), mBDNF, proBDNF and two doses of MGO modified proBDNF (MproBDNF). DAPI staining (white) of nuclei shows apoptotic morphology (white arrow). Immunostaining with anti βIIItubulin (red) show differentiation degree. **b)** Bars represent the quantification of apoptosis (by nuclei morphology), expressed as the mean of % apoptosis ± SD of the total nuclei. **c)** The quantification of differentiation is expressed as the mean of % differentiation (βIIItubulin immunoreactivity quantified by ImageJ, divided by the number of β III tubulin positive cells). Values represent the mean of three independent experiments. Scale bar =25 μm . ** *p* < 0,01, *** *p* < 0,001 two tailed Student’s “t” test

In order to test the effects of the AGE modifications of proBDNF induced by oxidative stress conditions on neuron survival and differentiation, we treated recombinant proBDNF in vitro with reactive oxygen species that react with Arg and Cys residues, resulting in the formation of intermolecular modifications and crossovers of the protein (see Methods section). This modified proBDNF (MproBDNF) was used to stimulate primary cultures of neurons along with mature BDNF, unmodified proBDNF as well as an unrelated protein, Bovine Serum Albumin (BSA), modified in the same way as the proBDNF (MBSA), as stimulation controls. In these experiments we used differentiated hippocampal neurons obtained from hippocampal NSCs plated on laminin-coated plates (see Methods). At 1 day in vitro (DIV), the neurons started to differentiate but it was between 4 and 7 DIV that the degree of maturation is significative. In longer culture periods, neurons displayed a complex degree of neuritogenesis and differentiation, mainly in the mBDNF treated wells, which was difficult to quantify. Differentiated neurons expressed doublecortin (DCX), βIIItubulin, SV2, p75, Sortilin, TrkB and calretinin (Fig. 3, Additional file 1: Figures S1-S3). These neurons, however, were negative for parvoalbumin and calbindin (Additional file 1: Figure S3), indicating that they are young granular differentiated neurons. In control cultures treated with mature BDNF for 6 DIV, neurons increased significantly their differentiation compared to unstimulated cultures, as demonstrated by β IIItubulin staining (Fig. 3a). Interestingly, control stimulation with proBDNF, without AGE modifications, did not increase apoptotic cell death over basal levels; in contrast, it produced similar effects on neuron differentiation as the mature BDNF (Fig. 3a, b). However, stimulation with MproBDNF, produced a significant increase of apoptosis and impaired neuron differentiation (Fig. 3a, b, c). These effects were specific for the AGE modified form of proBDNF (MproBDNF) since they were not observed upon stimulation with MGO modified BSA (MBSA) (Fig. 3a, b, c). These results suggest that proBDNF is quickly converted into the mature form in culture, inducing nearly the same effects as the BDNF, while the MproBDNF, which is more resistant to the action of convertases, is more stable and triggers the adverse effects associated with its pro-apoptotic activity.

### CSF from AD patients induces neuronal apoptosis through proBDNF/p75

**Fig. 4:**
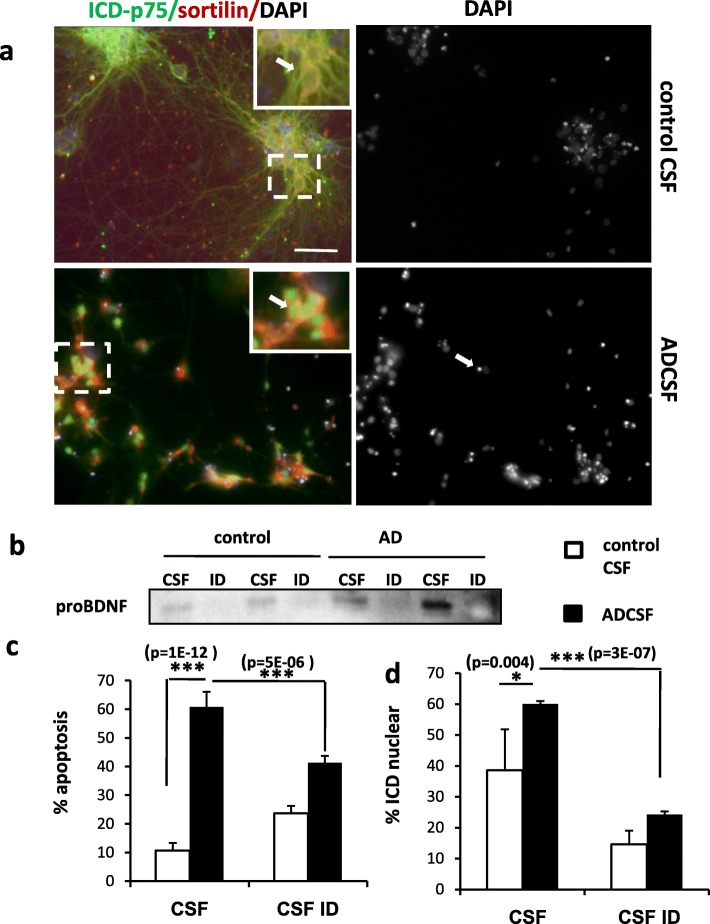
Human proBDNF from CSF AD patients induces apoptosis in neuronal culture. **a)** Representative pictures of neuronal primary culture from control mice Dentate Gyrus. Cells were treated for 6 days (7DIV) with human CSF from controls (control CSF) and from AD-affected patients (ADCSF). DAPI staining of nuclei shows apoptotic morphology (white arrow). Immunofluorescence with anti p75 ICD domain (green) and anti Sortilin (red). Inset indicate a higher magnification image of the boxed areas. White arrow in control CSF shows neurons with peripheral p75ICD location. White arrows in AD CSF show p75ICD nuclear location. **b)** Representative image of western blot showing the levels of proBDNF in CSF and in CSF immunodepleted with anti proBDNF (ID), from control and AD samples. **c and d)** Cells are treated with human 8 control CSF and 8 ADCSF, in both cases directly (CSF) or proBDNF immunodepleted (SCFID). **c)** Bars represent the quantification of apoptosis (by nuclei morphology), expressed as the mean of % apoptosis ± SD of the positive cells for both p75 and Sortilin. **d)** Bars represent the quantification of the mean of the % ± SD of neurons with the ICDp75 translocated to the nuclei (% ICD nuclear). Scale bar =50 μm . * *p* < 0,05, ** *p* < 0,01, two tailed Student’s “t” test

Our results have shown so far that in AD patients there is an increase of proBDNF/BDNF expression ratio as well as an increase of expression of some of the key signaling elements involved in the pro-apoptotic effects of this pro-neurotrophin. Moreover, we have observed that proBDNF from AD patients displays AGE post-traductional modifications as a consequence of the increase of the oxidative stress during neurodegeneration. Finally, our in vitro experiments on differentiated neurons suggest that these modifications produce a more stable form of proBDNF, a process which can account for 1) the increased levels of proBDNF in AD brains and CSF and 2) the contribution of proBDNF to the cell death associated with the disease. To evaluate further this hypothesis we directly evaluate the effects of the proBDNF contained in the CSF of AD patients and controls on differentiated primary neurons, as above. Thus, we stimulated the cultures for 6 DIV with CSF (substituting all the culture media, see Methods) from control and AD patients and stained the neurons with antibodies against p75ICD or with DAPI staining, in order to assess the subcellular localization of the protein by confocal microscopy and the apoptotic cell death, respectively (Fig. 4a and Additional file 1: Figure S3). Control CSF-treated neurons survived and differentiated normally showing a basal rate of apoptotic cell death of around 10% (Fig. 4a and c). A high proportion of these neurons displayed a peripheral distribution of p75 in the soma consistent with the presence of the intact receptor in the plasma membrane (Fig. 4a, c). In contrast, cultures treated with ADCSF showed an altered morphology with impaired differentiation and a dramatic increase the number of apoptotic nuclei (reaching 60%) (Fig. 4a, c). Moreover, under ADCSF treatment, there was a significant increase in the percentage of neurons with nuclear immunoreacitvity for p75, indicating that the 20 kDa ICD fragment was shed and translocated to the nucleus (Fig. 4a, d). The CSF from AD patients is known to contain several other factors, such as Au, that might be responsible for these neurotoxic effects. To determine whether proBDNF present in the CSF of AD patients we specifically immunodepleted the CSF samples from proBDNF with specific anti-proBDNF antibodies coupled to sepharose AG beads. Successful depletion was demonstrated by Western blot with antibodies against proBDNF (Fig. 4b). We then compared the effects of the immunodepleted CSF (CSFID) and the normal CSF from both, AD patients and controls, in our neuron cultures after 6 DIV. As shown in Fig. 4c and d, depletion of proBDNF produced a remarkable reduction of the percentage of apoptosis as well as the percentage of neurons with nuclear immunoreactivity for p75. Therefore, these results indicate that the proBDNF in the CSF of AD patients is biologically active and that it is the main responsible of the pro-apoptotic effects triggered by AD CSF. Moreover, these results strongly suggest that the effects of proBDNF in AD CSF are mediated by activation of p75 signaling.

### APP/PS1 mutations increase p75 processing and apoptosis induction

**Fig. 5:**
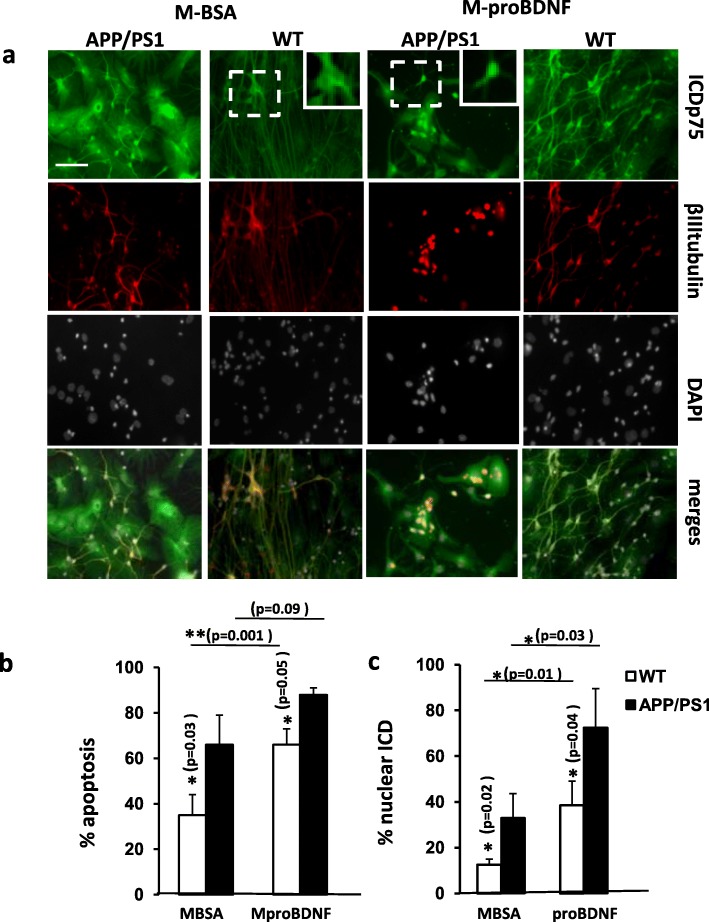
Apoptosis and p75 processing are increased in neuronal cultures from APP/PS1 animals. Primary cultures of differentiated mouse hippocampal NSCs were treated for 6 days (7DIV) with MGO modified BSA (MBSA) or with MGO modified proBDNF (MproBDNF). **a)** Representative immunofluorescence pictures of cells from WT and APP/P1 mice labeled with ICDp75 (green), βIIItubulin (red) and DAPI (white). Inset represents a higher magnification image of the boxed areas indicating citoplasmatic (WT) and nuclear localization (APP/PS1) of ICDp75. **b)** Bars represent the quantification of apoptosis (by nuclei morphology), expressed as the mean of % apoptosis ± SD of the positive cells for both ICDp75 and βIIItubulin. **c)** Bars represent the mean of the % ± SD of neurons with the ICDp75 translocated to the nuclei (% ICD nuclear). The values represent the mean of three independent experiments Scale bar =50 μm . ** *p* < 0,01, two tailed Student’s “t” test

In the familiar AD, one of the most frequent targets of inherited mutations is the presenilin-gamma secretase complex [37]. Some of these mutations affect mainly presenilin 1 (PS1) including the deletion of exon 9 (u9) or M146 V [38]. These are supposed to be gain of function mutations leading to an increase of protease activity [39]. The study of the pathogenic mechanisms underlying these mutations and the development of AD has been focused on several of the PS1 substrates, mainly Aβ [39]. Since p75 is also a substrate of PS1 activity [18, 40], we hypothesized that the harmful consequences of proBDNF on neuron survival and differentiation in AD, due to its higher stability as a consequence of AGE modifications, could be boosted by the effects of PS1 mutations on p75 signaling. In particular, we were interested in ascertain whether the shedding of p75, which requires PS1 activity, changes during AD and whether these changes can sensitize the effects of pro-neurotrophins. To test this hypothesis we set up primary neuron cultures from NSCs from heterozygous APP/PS1 mice or from wild type littermates as controls (see Methods). This APP/PS1 animal model has been widely used in studies of neurological disorders of the brain, specifically AD, amyloid plaque formation, and aging [41]. After 6 DIV, when all the conditions are considered for the analysis (regardless to the treatment), APP/PS1 cultures does not show a % of apoptosis significantly different than WT (One-way ANOVA *p* = 0.1). However under treatment with MBSA (control), these transgenic APP/PS1 neurons displayed higher percentage of apoptotic nuclei and higher percentage of neurons with nuclear immunoreactivity for p75 (Fig. 5a, b, c and Additional file 1: Figure S3). Interestingly, stimulation with MproBDNF induced a stronger and significant increase in cell death and p75 nuclear immunoreactivity in these transgenic neurons compared to controls (Fig. 5a, b, c). We therefore conclude that PS1 mutations involved in familiar AD potentiate the effects of proBDNF on cell death most probably by increasing p75 signaling and, in particular, p75ICD nuclear translocation.

## Discussion

The pathogenic mechanisms underlying the development of AD are complex and yet not fully understood. Some important research lines have focused on the role of neurotrophins in AD both, as regulating factors of cell survival and cell death, involved in the pathogenesis and/or the course of the disease, and as therapeutic tools [2, 3]. Today, most of our knowledge about the role of neurotrophins in AD comes from the study of the NGF and its precursor form, proNGF. Thus, proNGF has been reported to be increased during aging and AD in vulnerable regions such as the hippocampus [42]. Moreover, the proNGF isolated from AD patients displays higher neurotoxicity than proNGF from controls [27]. More controversial is the role of proBDNF/BDNF alterations in the pathogenicity of the AD. In part, because contradictory evidences were reported regarding the expression and secretion of BDNF in its precursor form [6, 43, 44]. It was suggested that these opposing results reflect a more complex regulation of proBDNF processing that depends on the regulation by third-parties such as glial cells and plasmin inhibitors [45]. Indeed, BDNF is more important for the maintenance and function of central nervous system than NGF and thus, reduced levels of BDNF, due to impairment of proBDNF processing, could be involved in some aspects of the AD disease, such as depression, in addition to the neurotoxic effects of the pro-neurotrophin [46].

In the present work we have addressed the role of proBDNF and its pro-apoptotic signaling mechanisms in AD. We showed that in brains from AD patients there is an increase of the levels of proBDNF are increased, as are also some of the key signaling components involved in its pro-apoptotic effects, such as the co-receptor Sortilin. We were also able to detect an increase of proBDNF in the CSF of AD patients. Interestingly, this increase of proBDNF in the CSF is associated with a decrease of BDNF. Moreover, we have demonstrated that the proBDNF in the CSF from AD patients is highly modified by oxidative stress and that the presence of this AGE modified proBDNF induces apoptosis and impairs differentiation on primary cultures of neurons. Finally, we have shown that the damaging effects of proBDNF can be potentiated by mutations commonly found in familiar forms of AD that affects PS1 protease activity. This synergistic effect is due to the enhancement of p75 signaling, in particular of p75ICD internalization. The present results further reveal the important role of neurotrophin signaling in the development of the AD and, in particular, our results provide evidence for the relevance of the proBDNF/p75 pathway in the pathogenesis and/or the course of AD. Thus, these results highlight the importance of proBDNF/p75 signaling as potential targets to develop novel pharmacological therapies to fight AD.

During aging and especially during neurodegeneration there is an increase of oxidative stress in the tissue that contributes to the pathogenesis of AD [1]. The neurotoxic mechanisms involved, however, are not clearly understood. Glyoxal (GO) and methylglyoxal (MG) are highly reactive dicarbonyl formed during the metabolism that, in excess, can increase ROS production and cause oxidative stress. GO, and MGO can react with free amino groups of Lys, Arg and Cys residues, leading to the formation of the AGE/ALE adducts CML, CEL and intermolecular crosslink [47]. The relationship between protein carbonylation and neurodegeneration (ND) has been widely established. Changes in protein carbonyl samples from both disease experimental animal models and patients with neurodegeneration have been reported [48, 49]. Moreover, mass spectrometry analysis of human brain homogenates has shown that several AGE/ALE adducts, HNEL, CEL, CML and Nε-(malondialdehyde)-lysine (MDAL) increase in ND [1, 35]. Oxidative non-enzymatic modifications can also affect protein structure and function by other means, for example increasing protein crosslinking and thus contributes to protein aggregation during ND [1]. These general effects on protein structure might affect the function of some proteins in a specific manner. In the present work, we found that proBDNF co-localizes almost entirely with CEL in AD samples and that the proBDNF isolated from the CSF of AD patients display higher CEL modifications than controls. We therefore suggest that the increase of proBDNF in AD patients, in brain samples and in CSF, is due to a higher stability of this precursor form resulting from post-traductional modifications triggered by oxidative stress that make this modified pro-neurotrophin more resistant to be processed by convertases into the mature BDNF. The target sequence for Furin and other convertases (plasmin and metalloproteases) contains Lys, Arg, and Cys residues that are indeed suitable sites for AGE/ALEs formation [50]. Similar results were obtained by our group when we analyzed the role of proNGF/NGF in human hippocampal samples from AD patients [16, 27, 28]. We cannot rule out the possibility that AGE modified proBDNF could have acquired gain-of-function effects independent of proBDNF signaling. Nevertheless, since there is a strong correlation between proBDNF levels in AD and p75 signaling (see below) we rather favor the hypothesis these oxidative stress-induced modifications makes the proBDNF more stable and tip the balance in favour of cell death (increase of proBDNF) at expenses of cell survival (decrease of BDNF). Accordingly, we observed in the CSF of AD patients an increase of proBDNF that was associated with a decrease of BDNF. In a more functional approach we demonstrated that the CSF from AD patients, but not the CSF from controls, induces apoptosis in a primary culture of differentiated neurons obtained from NSCs from the Dentate Gyrus. In contrast, CSF from control individuals maintained the survival and differentiation of these neurons up to 6 DIV. Interestingly, depletion of proBDNF from AD CSF samples reduced strongly the apoptotic effect of the original AD CSF (apoptosis reduced from 40 to 20%), suggesting that proBDNF is a main factor that triggers the apoptotic effect of the AD CSF. The remaining 20% apoptosis could be due to other pro-apoptotic inducers such as Aβ or proNGF (references). Finally, we propose that the ratio proBDNF/BDNF measured in the in the CSF could be a potential diagnostic marker for AD. To further validate this approach, it would be interesting to measure the proBDNF/BDNF ratio in the CSF of a larger cohort of patients and controls and analyze this ratio in regard to the different stages of the disease.

BDNF genetic variation may affect the risk of developing AD [51]. One of these variations, proBDNF^Val66Met^, impairs the normal processing of proBDNF resulting in higher levels of expression of proBDNF [52]. The Val66Met polymorphism affects a particular step of proBDNF processing that is sufficient to impair the formation of enough levels of mature BDNF and trigger familiar depression [46]. The mutation blocks proBDNF maturation and affects activity-dependent secretion of BDNF and human memory and hippocampal function [53, 54] and worsens vulnerability to stress and response to antidepressants [55]. Despite the proBDNF^Val66Me^ is able to interact with p75 and Sortilin, altering the neuronal morphology [52], a recent meta-analysis did not show an association of the Val66Met polymorphism and the risk of AD [56]. It is possible that the proBDNF^Val66Met^ is still partially processed reducing the neurotoxic effects of the full proBDNF form.

Besides the increase of proBDNF levels in AD patients, we provide evidence for the existence of at least two additional mechanisms that could potentiate the neurotoxic action of proBDNF and accelerate the disease. First, we observed that the levels of Sortilin, the co-receptor of p75NTR involved in the pro-apoptotic effect of proBDNF, are increased in the hileal region of the hippocampus of AD patients thus making the neurons of this region more vulnerable to cell death. Sortilin levels seem to be particularly sensitive to ageing and neurodegeneration. For instance, Sortilin immunoreactivity was described to be increased in ageing rodent basal forebrain and sympathetic neurons while the levels of p75NTR were either unchanged or reduced [57]. On the other hand and similarly to our results, Sortilin was shown to be increased in the temporal cortical area of human AD brains as well as in brains of 6-months old PS1delta9 transgenic mice [58]. Interestingly, this increase was suggested to be triggered by amyloid beta_1_-_42_ (Aβ_42_) through the p75/RhoA signaling pathway suggesting a potential physiological interaction of Aβ_42_, p75NTR and sortilin in AD [57]. From all this data we propose that neurodegeneration in AD patients may be enhanced by an increase of sortilin expression in neurons, probably induced by Aβ_42_, together with elevated levels of proBDNF in specific regions.

Secondly, we found that proBDNF effects in AD can also be enhanced by mutations found in familiar AD that affect the convertase activity of the γ-secretase PS1. We propose that these mutations make neurons more sensitive to the pro-apoptotic effect of proBDNF by increasing the susceptibility of p75 to be processed and induce nuclear translocation of the p75ICD. Although in familiar AD PS1 mutations are described to increase Aß42/40 ratio, the effect of these mutations on its convertase activity as gain or loss of function is controversial [39]. In the present work, we have observed an increase of basal cleavage of p75 and an increase of p75ICD nuclear translocation and apoptosis (further induced by MproBDNF), in neurons from the animal model of AD APP/PS1 bearing the mutation ∆9 in PS1. Therefore we favor the idea that, at least the ∆9 deletion in PS1, behaves as a gain of function mutation.

More than fifty membrane proteins, including APP and p75, are cleaved by the presenilin1- γ-secretase complex [59, 60]. APP processing and Aβ production is one of the hallmarks of AD [29]. On the other hand, the cleavage of p75 and the production of the ICD fragment that is translocated to the nucleus, is considered a key step during the apoptotic signaling pathway triggered by this receptor although the exact physiological effects of p75ICD release depends on the neuronal subtype [18, 40]. Recently, experimental evidence indicates that p75 signaling, including cleavage and release of the ICD, is indeed strongly related to AD, highlighting the central role of p75 during AD pathogenesis [59]. Thus, several mechanisms have been proposed involving p75 signaling in AD. For instance, it was shown that Aβ is able to bind to p75 and trigger activation of the receptor inducing the cleavage of the ICD [60]. Moreover, amyloidogenesis is aggravated by the interaction of beta-site amyloid precursor protein cleaving enzyme-1 (BACE1) with p75 upon the binding of Aβ to p75 [21]. In contrast, it was shown that the p75ECD has a neuroprotective effect against Aβ toxicity [61] and that transmembrane domain of p75, has trophic effects by inducing phosphorylation of TrKB [62]. Our results emphasize the important contribution of p75 signaling, including p75ICD internalization and apoptosis, in AD pathogenesis upon AGE-modified proBDNF stimulation. More importantly, our data provide a novel pro-apoptotic mechanism in AD and involving p75 in which higher levels of cleavage due to mutations in PS1 make these neurons more sensitive to the adverse effects of AGE-modified proBDNF. Moreover, we suggest that this is a general mechanism in AD, also for the non-familiar, spontaneous form of the disease, given the fact that Aβ is able to trigger p75 cleavage and p75ICD release and could therefore prime p75 signaling for AGE modified proBDNF independenty of PS1 mutations.

## Additional file

Additional file 1: **Figure S1.** NSCs in culture express p75NTR, sortilin, DCX and SV2. **Figure S2.** NSCs in culture expressing DCX and SV2, are positives for TrkB. **Figure S3.** Characterization of Adult NSCs in culture. **Figure S4.** IDC p75NTR cellular distribution in NSC. (PDF 1196 kb)

## Abbreviations

AD: Alzheimer disease
AGEs: ROS derived advanced glycation end products
ALEs: Advanced lipooxidation end products
BACE1: α-protease
BDNF: Brain-derived neurotrophic factor
BSA: Bovine Serum Albumin
CEL: Nε-(carboxyethyl)-lysine
CML: Carboxymethyl)-lysine
CSF: Cerebrospinal fluid
CSFID: Immunodepleted CSF
DIV: Day in vitro
DMEM: Dulbecco’s modified Eagle’s medium
EGF: Epidermal Growth Factor
ELISA: Enzyme-immuno assay
FGF: Fibroblast Growth Factor
GO: Glyoxal
HNEL: 4-hydroxy-2-nonenal-lysine
ICD: Intracellular domain
MBSA: MGO-modified BSA
MDAL: Nε-(malondialdehyde)-lysine
MGO: methylglyoxal
MMSE: Minimental state examination
MproBDNF: MGO-modified proBDNF
ND: Neurodegeneration
NSCs: Hippocampal Neural Stem Cells
OS: Oxidative stress
P/S: Penicylin/Streptomycin
PBS: Phosphate buffered saline
PBS-T: PBS with 0, 1% Triton X-100
PFA: Paraformaldehyde
pH 8.0: 133 mM NaCl, 0.2% Tween 20
proBDNF: Precursor form of BDNF
PS1: Presenilin 1
ROS: Radical Oxygen Species
SD: Standard deviation
TBS-T: 50 mM Tris
