# Supplementary material for: proBDNF is modified by advanced glycation end products in Alzheimer’s disease and causes neuronal apoptosis by inducing p75 neurotrophin receptor processing
Source: Mol Brain. 2018 Nov 14;11:68. doi: 10.1186/s13041-018-0411-6 (PMC6237019; doi:10.1186/s13041-018-0411-6)

Figure S1 NSCs in culture express p75NTR, sortilin, DCX and SV2.

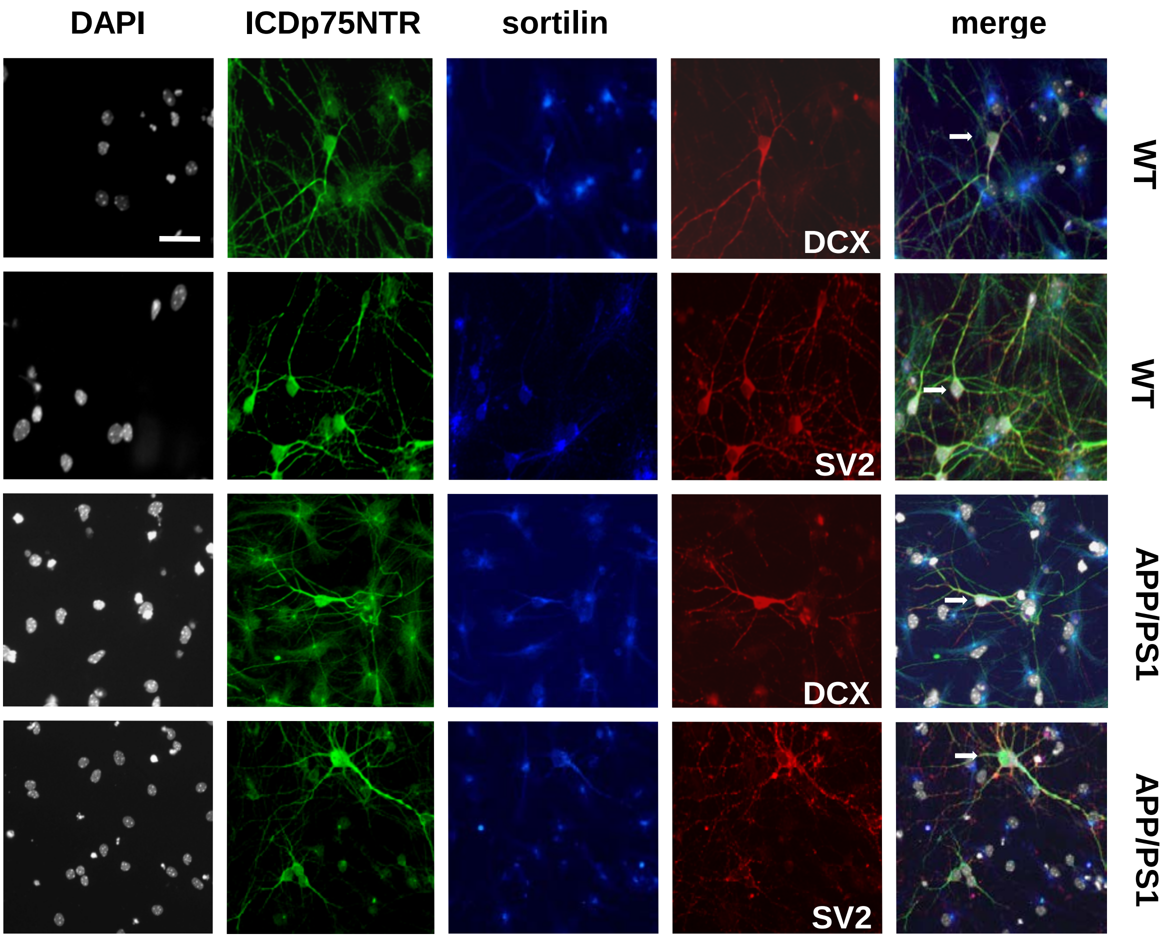

Figure S2 NSCs in culture expressing DCX and SV2, are positives for TrkB

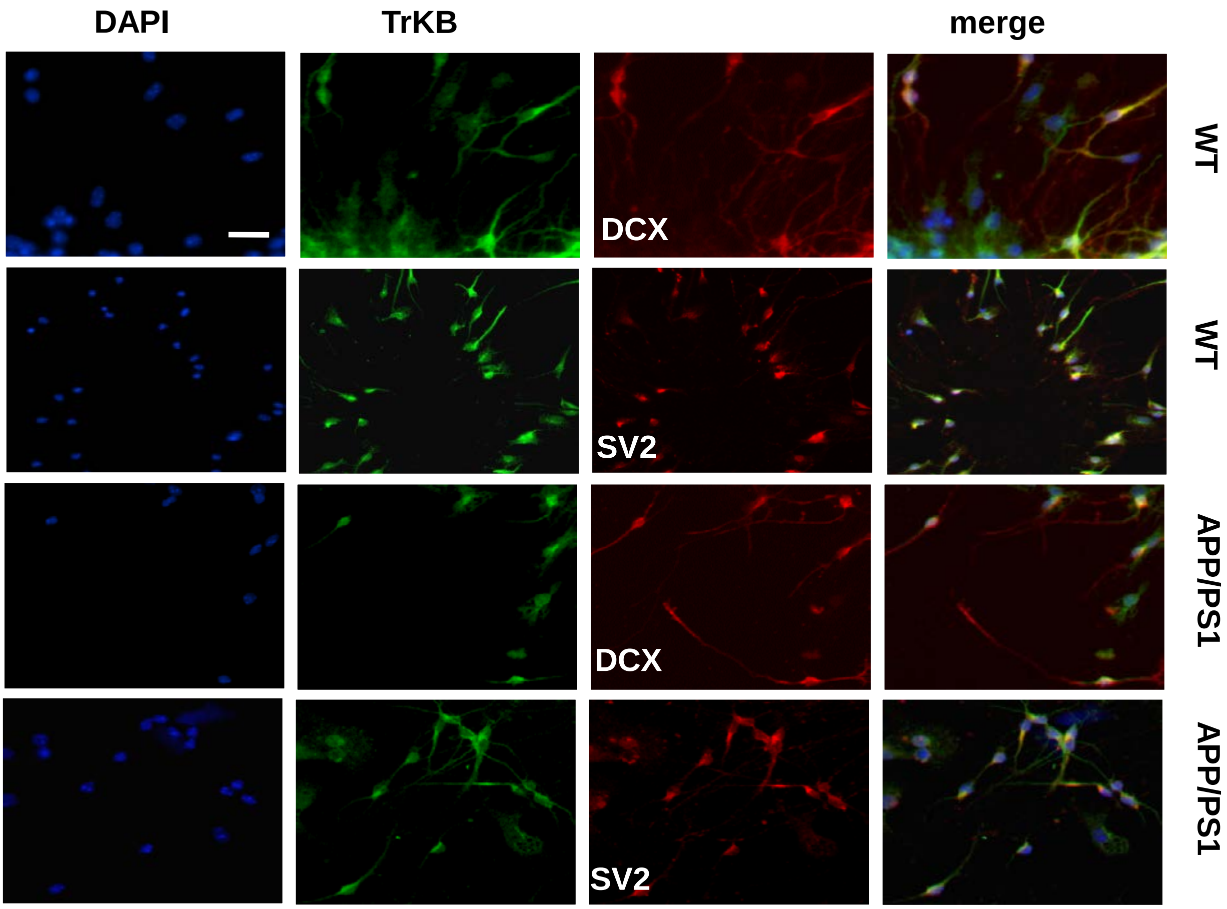

Figure S3 Characterization of Adult NSCs in culture.

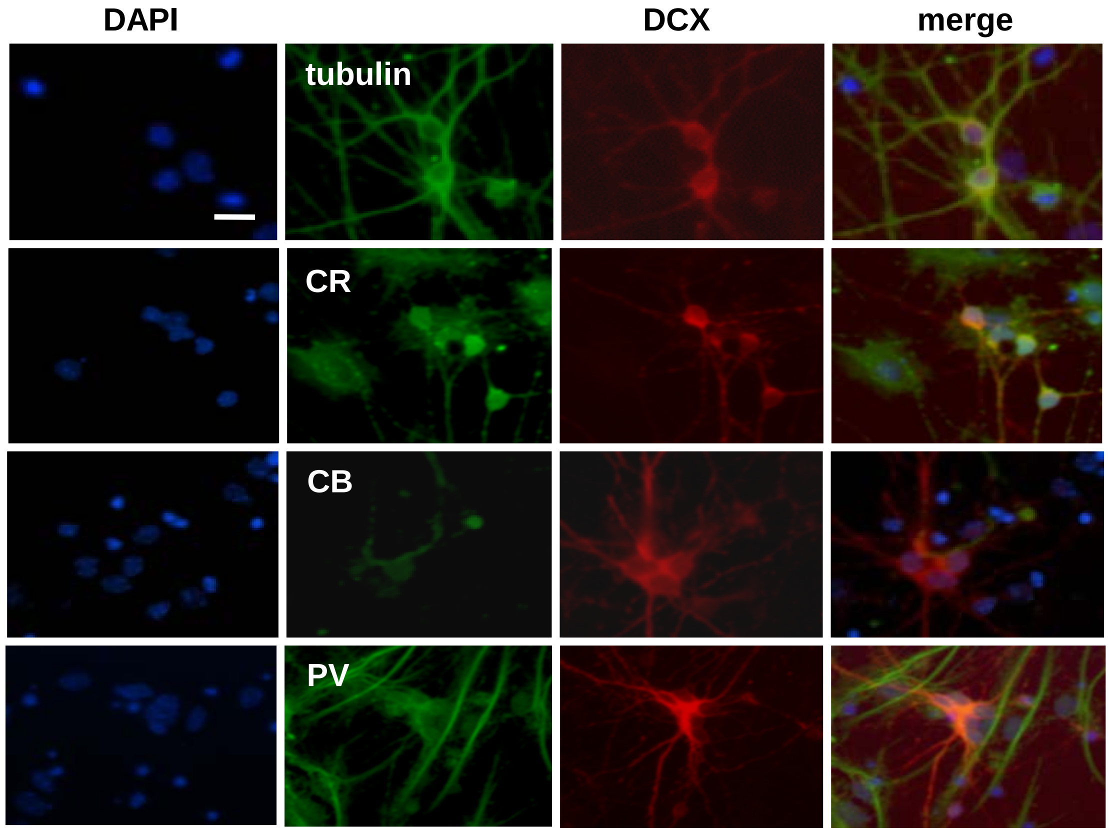

Figure S4 IDC p75NTR cellular distribution in NSC.

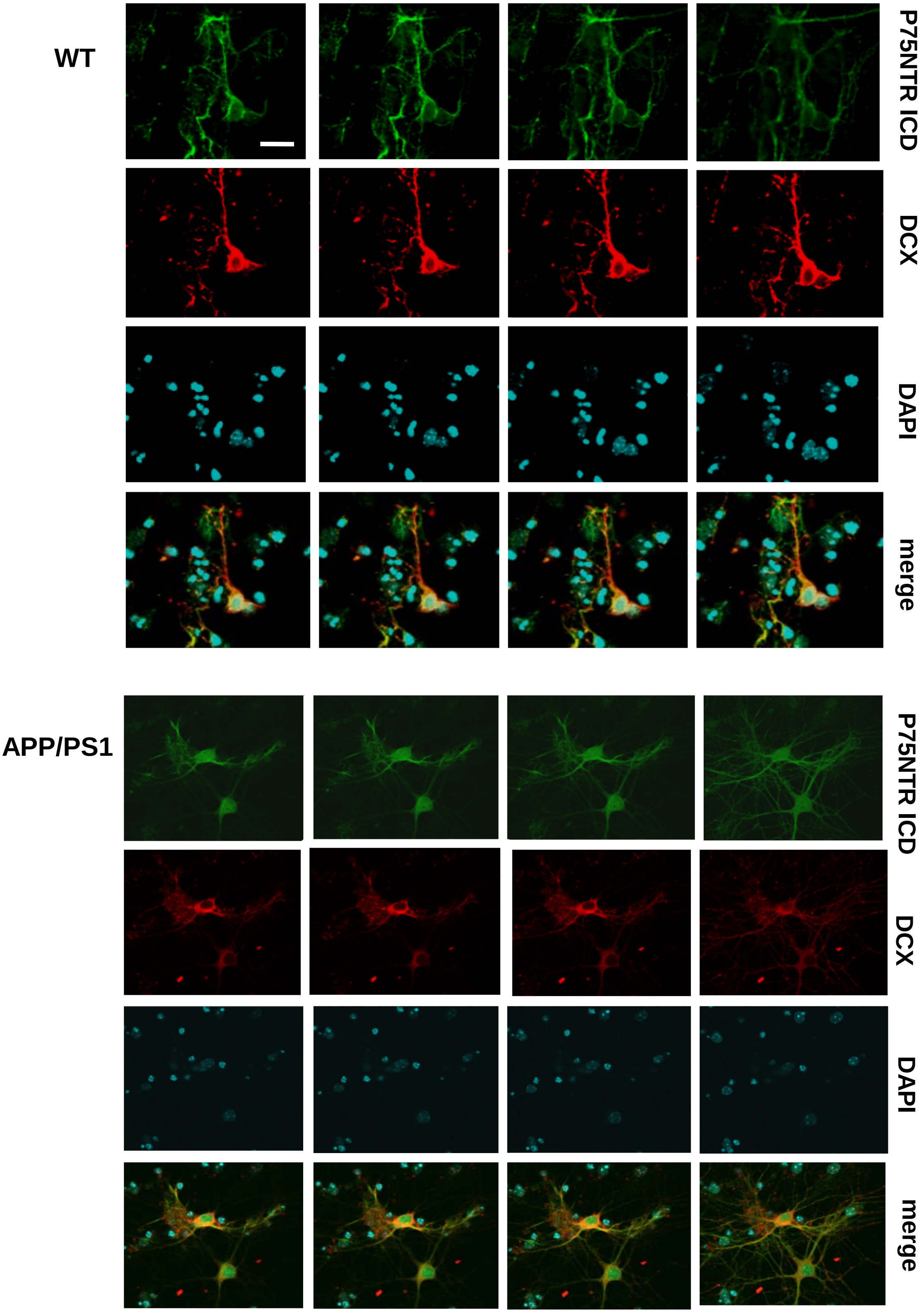

Supplement: Supplementary file 1 — Figure S1. NSCs in culture express p75NTR, sortilin, DCX and SV2. Figure S2. NSCs in culture expressing DCX and SV2, are positives for TrkB. Figure S3. Characterization of Adult NSCs in culture. Figure S4. IDC p75NTR cellular distribution in NSC. (PDF 1196 kb) [file 13041_2018_411_MOESM1_ESM.pdf]
